# Clinical characteristics in blood stream infections caused by *Klebsiella pneumoniae*, *Klebsiella variicola,* and *Klebsiella quasipneumoniae*: a comparative study, Japan, 2014–2017

**DOI:** 10.1186/s12879-019-4498-x

**Published:** 2019-11-08

**Authors:** Kazuo Imai, Noriomi Ishibashi, Masahiro Kodana, Norihito Tarumoto, Jun Sakai, Toru Kawamura, Shinichi Takeuchi, Yoshitada Taji, Yasuhiro Ebihara, Kenji Ikebuchi, Takashi Murakami, Takuya Maeda, Kotaro Mitsutake, Shigefumi Maesaki

**Affiliations:** 1 Department of Infectious Disease and Infection Control, Saitama Medical University, 38 Morohongo, Moroyama-machi, Iruma-gun, Saitama, 350-0495 Japan; 2 Center for Clinical Infectious Diseases and Research, Saitama Medical University, 38 Morohongo, Moroyama-machi, Iruma-gun, Saitama, 350-0495 Japan; 3 Infectious Diseases and Infection Control, Saitama Medical University International Medical Center, 1-1397 Yamane, Hidaka, Saitama, 350-1298 Japan; 4 Clinical Laboratory Medicine, Saitama Medical University Hospital, 38 Morohongo, Moroyama-machi, Iruma-gun, Saitama, 350-0495 Japan; 5 Department of Clinical Laboratory Medicine, Saitama Medical University International Medical Center, 1-1397 Yamane, Hidaka, Saitama, 350-1298 Japan; 6 Department of Microbiology, Saitama Medical University, 38 Morohongo, Moroyama-machi, Iruma-gun, Saitama, 350-0495 Japan

**Keywords:** *Klebsiella pneumoniae*, *Klebsiella variicola*, *Klebsiella quasipneumoniae*, Blood stream infection, Japan

## Abstract

**Background:**

*Klebsiella variicola* and *K. quasipneumoniae* are new species distinguishable from *K. pneumoniae* but they are often misidentified as *K. pneumoniae* in clinical settings. Several reports have demonstrated the possibility that the virulence factors and clinical features differ among these three phylogroups. In this study, we aimed to clarify whether there were differences in clinical and bacterial features between the three phylogroups isolated from patients with bloodstream infections (BSIs) in Japan.

**Methods:**

Isolates from all patients with BSIs caused by *K. pneumoniae* admitted to two hospitals between 2014 and 2017 (*n* = 119) were included in the study. Bacterial species were identified via sequence analysis, and their virulence factors and serotypes were analyzed via multiplex PCR results. Clinical data were retrieved from medical records.

**Results:**

Of the 119 isolates, 21 (17.7%) were identified as *K. variicola* and 11 (9.2%) as *K. quasipneumoniae*; K1 serotype was found in 16 (13.4%), and K2 serotype in 13 (10.9%). Significant differences in the prevalence of *rmpA*, *iutA*, *ybtS*, *entB* and *kfu* (*p* < 0.001), and *allS* genes (*p* < 0.05) were found between the three phylogroups. However, there were no significant differences in clinical features, including the 30-day mortality rate, between the three organisms, although *K. variicola* was more frequently detected in patients over 80 years old compared with other *Klebsiella* species (*p* < 0.005), and *K. quasipneumoniae* more frequently occurred in patients with malignancy (*p* < 0.05).

**Conclusions:**

Our findings demonstrated the differences in bacterial pathogenicity and clinical features among these three phylogroups. Further epidemiological studies into BSI caused by *Klebsiella* species are warranted.

## Background

*Klebsiella pneumoniae* is a frequent cause of infectious diseases in hospitals and community settings and is associated with a wide variety of clinical conditions including pneumonia, intra-abdominal infections, urinary tract infections, and bloodstream infections (BSIs). *K. pneumoniae* is reported to be the second most common cause of Gram-negative bacteremia and has a high mortality rate [1].

Recently, various microbiological factors and virulence genes of *K. pneumoniae* have been reported to be associated with the clinical features and a high mortality rate. Regarding the phenotypic features, it was demonstrated that a hypermucoviscous phenotype of *K. pneumoniae* could be a contributing factor in community-acquired primary liver abscesses [2, 3]. The hypermucoviscous phenotype was previously common primarily in Asian countries, but it is now common worldwide [4]. Most isolates of the hypermucoviscous phenotype belong to the capsular K1 and K2 serotypes, and the predominant underlying mechanisms are due to the presence of regulator of mucoid phenotype A (*rmpA*) and plasmid-borne regulator of extracellular polysaccharide synthesis (*magA*) [5–7]. Additionally, iron-scavenging systems (such as enterobactin, yersiniabactin, aerobactin [8, 9], and *kfu* ion uptake system [10]), pili (type 3 fimbrial adhesin protein) [11], and allantoin metabolism [12] can also contribute to the virulence and the pathogenicity of *K. pneumoniae*.

The taxonomy of *Klebsiella* genus has been extensively studied, and *K. variicola* [13] and *K. quasipneumoniae* [14] were distinguished from *K. pneumoniae* as new species in 2004—previously, they were classified as members of *K. pneumoniae* phylogroups KpIII and KpII, respectively [13, 14]. However, it is difficult to discriminate between these species using conventional laboratory methods because of their close phenotypic and biochemical features [15–17]. Recently, direct bacterial profiling via matrix-assisted laser desorption ionization–time of flight mass spectrometry (MALDI-TOF MS) has been established as a tool for the rapid identification of bacteria [18]. However, mass spectra derived from *K. variicola* and *K. quasipneumoniae* are very similar to the spectrum of *K. pneumoniae*. Therefore, these species have been erroneously identified as *K. pneumoniae* in clinical settings [17].

Maatallah and colleagues have reported that patients with *K. variicola* BSIs should be considered to be at higher risk of mortality than those with *K. pneumoniae* or *K. quasipneumoniae* BSIs [19]. However, few reports have evaluated the differences and the clinical impact between these three organisms; hence, further clinical investigation is needed.

In this study, we aimed to clarify differences in the clinical impact and bacterial characteristics of BSIs associated with *K. pneumoniae*, *K. variicola*, and *K. quasipneumoniae* in Japan from 2014 to 2017.

## Methods

### Patients and bacterial isolates

All patients with *K. pneumoniae* detected in blood samples who were admitted to Saitama Medical University Hospital and Saitama Medical University International Medical Center—a 1000-bed and a 700-bed tertiary care hospital and referral center, respectively—in Saitama, Japan, from 2014 to 2017 were included in this study. Only the first episode of bacteremia in each patient was included in this retrospective analysis, and 119 patients were finally enrolled. All isolates derived from blood cultures were identified by a MALDI Biotyper with MALDI Biotyper 3.1 software and MALDI Biotyper Reference Library version 4.0.0.1 (Bruker Daltonics, Bremen, Germany) according to the manufacturer’s instructions in an autoflex speed mass spectrometer (Bruker Daltonics), which could not discriminate between *K. variicola, K. quasipneumoniae,* and *K. pneumoniae* [17]*,* and stored at − 80 °C. Antimicrobial susceptibility testing was performed by Microscan Walk Away 96 Plus (Beckman Coulter, Brea, CA) and potential extended spectrum beta-lactamase (ESBL) producers were further tested and confirmed using a combined disk test according to the Clinical and Laboratory Standards Institute (CLSI) guidelines (document M100-S27).

### Clinical parameters

A retrospective cohort study was conducted to evaluate the risk factors for mortality among the patients; 30-day mortality was the primary outcome measurement in this study. We collected patient information retrospectively from the electronic medical records of the hospitals. The data collected included the following: age, sex, patient risk factors (underlying disease, use of immunosuppressant and steroids, hemodialysis, and neutropenia; neutrophil count < 500 cells/m^3^), source of infection (which was reviewed retrospectively and described as “unknown” if not described in the medical records or determined by the attending physicians), polymicrobial bacteremia, use of catecholamines for septic shock, hospital-acquired infection versus community-acquired infection (infections were defined as hospital-acquired if the blood cultures were collected > 48 h after admittance to hospital or if the patient had been admitted to hospital within the previous 30 days), and appropriate or inappropriate antibiotic therapy within 24 h from the first BSI episode. Appropriate antibiotic therapy was defined as use of an antimicrobial agent to which isolates were susceptible based on in vitro susceptibility testing.

### Identification of *K. variicola* and *K. quasipneumoniae* from *K. pneumoniae* isolates

The isolates, which were initially identified as *K. pneumoniae* using MALDI-TOF MS and stored until use, were identified by sequence analysis of subunit C of topoisomerase IV (*parC*) gene. Primer pairs for *parC* gene were used to identify *K. variicola* and *K. quasipneumoniae* according to previously reported protocols [20]. All isolates were cultured on Mueller-Hinton agar in advance. To prepare the template DNA for PCR, bacterial cells from a single colony were suspended in sterile distilled water and lysed at 98 °C for 5 min. The lysates were centrifuged at 13,000×*g* for 5 min and 1 μL of supernatant was used for PCR reactions. Briefly, PCR amplicons were generated by TaKaRa Ex Taq (Takara Bio Inc., Gunma, Japan) and thermal cycling was carried out under the following conditions: 94 °C for 5 min, followed by 30 cycles at 98 °C for 10 s, 53 °C for 30 s, 72 °C for 30 s, with a final extension at 72 °C for 5 min. PCR products were analyzed using 1.2% (w/v) agarose gel stained with ethidium bromide and purified using by ExoSAP-IT Express PCR Product Cleanup Reagent (Thermo Fisher Scientific, Massachusetts, USA). Sanger sequencing of these purified PCR amplicons was performed by Eurofines Genomics Co., Ltd. (Tokyo, Japan). Phylogenetic analysis was performed by MEGA 6 (https://www.megasoftware.net/). The phylogenetic tree was constructed by the neighbor-joining method based on the analysis of 319 bp of the *parC* gene of 119 clinical isolates and reference strains. *Klebsiella* species were determined by identifying the cluster of the reference strains to which they belonged in the phylogenetic tree.

The *parC* gene sequence of reference strains were obtained from NCBI database (GenBank accession numbers: *K. pneumoniae*, CP009208, CP026586, NZ_AOCV00000000, NZ_CP015134 and NZ_CP030269; *K. variicola*, NZ_CP017289 and NZ_CP030173; and *K. quasipneumoniae*, CP014696).

The reliability of the topology of the tree was checked by 500 bootstrap replications.

### Detection of virulence genes and serotyping

To determine the capsular serotypes (K1 and K2) and to clarify the presence of genes associated with virulence factors (*rmpA*, *entB*, *ybtS*, *iutA, kfu*, *mrkD*, and *allS*), we used Multiplex-PCR, according to previously reported protocols [21]. *entB, ybtS, iutA*, *and mrkD* encode enterobactin, yersiniabactin, aerobactin siderophore receptor, and the type 3 fimbrial adhesin protein, respectively. Furthermore, *kfu* and *allS* are associated with the iron uptake system and allantoin metabolism, respectively. PCR amplicons were generated by KOD Multi-Epi (Toyobo, Osaka, Japan) and thermal cycling was carried out under the following conditions: 94°C for 5 min, followed by 30 cycles at 98 °C for 10 s, 60°C for 30 s, 68 °C for 1 min, with a final extension at 68 °C for 5 min. PCR products were analyzed using 2% (w/v) agarose gel stained with ethidium bromide.

### String test

The hypermucoviscous phenotype of the isolates was determined using the string test, in which a standard bacteriological loop is used to stretch a mucoviscous string from each bacterial colony cultured on 5% sheep blood agar. The formation of a viscous string > 5 mm in length was regarded as a positive test result [3].

### Statistical analysis

Univariate analysis was performed to identify the differences in clinical characteristics between the three organisms. Univariate analysis was conducted using Fisher’s exact test. Factors considered in univariate analysis were included in the multivariate analysis. Multivariable logistic regression analysis was used to assess the association between independent risk factors and mortality. All statistical analyses were calculated with R (v 3.4.0; R Foundation for Statistical Computing, Vienna, Austria [http://www.R-project.org/]). A *p*-value of <0.05 was considered statistically significant.

## Results

### Bacterial characteristics

The characteristics of the isolates and the prevalence of virulence genes were characterized in Table 1. Of the 119 isolates previously identified as *K. pneumoniae* by MALDI-TOF MS, 21 (17.7%) were identified as *K. variicola* and 11 (9.2%) as *K. quasipneumoniae,* based on sequence analysis of the *parC* gene (Additional file 1: Figure S1). Of the 119 isolates, 25 (21.0%) were hypermucoviscous phenotype (*K. pneumoniae* = 22 *K. variicola* = 2, *K. quasipneumoniae* = 1). The prevalence of *rmpA* and K1 and K2 serotypes was significantly higher in the hypermucoviscous phenotype isolates than in the non-hypermucoviscous phenotype isolates (*rmpA*: 76.0% vs 10.6%, *p* < 0.001; K1: 32.0% vs 8.5%, *p* < 0.005; K2: 28.0% vs 6.3%, *p* < 0.005) (Additional file 2: Table S2). Isolates of the hypermucoviscous phenotype belonging to *K. variicola and K. quasipneumoniae* were not K1 or K2 serotypes and did not have *rmpA* gene (Additional file 2: Table S1).

**Table 1:** Summary of bacterial characteristics for *K. pneumoniae*, *K. variicola*, and *K. quasipneumoniae* isolates

	Total = 119		*K. pneumoniae* = 87		*K. variicola* = 21		*K. quasipneumoniae* = 11		*p*-value	
	n	%	n	%	n	%	n	%		
K1	16	(13.5)	15	(17.2)	0	(0)	1	(9.1)	0.09	
K2	13	(10.9)	12	(13.8)	1	(4.8)	0	(0)	0.32	
*rmpA*	29	(24.4)	29	(33.3)	0	(0)	0	(0)	<0.001	*
*iutA*	32	(26.9)	32	(36.8)	0	(0)	0	(0)	< 0.001	*
*ybtS*	45	(37.8)	41	(47.1)	4	(19.0)	0	(0)	<0.001	*
*entB*	108	(90.8)	85	(97.7)	19	(90.5)	4	(36.4)	< 0.001	*
*kfu*	45	(37.8)	26	(29.9)	19	(90.5)	0	(0)	< 0.001	*
*allS*	28	(23.5)	21	(24.1)	1	(4.8)	6	(54.5)	< 0.01	*
*mrkD*	118	(99.2)	86	(98.9)	21	(100.0)	11	(100.0)	1.00	
Hypermucoviscous phenotype	25	(21.0)	22	(25.3)	2	(9.5)	1	(9.1)	0.24	
ESBL-producing	3	(2.5)	2	(2.3)	1	(4.8)	0	(0)	0.61	

*Asterisk indicates a statistical significant difference among the groups

Regarding the prevalence of the 7 virulence genes, *rmpA* and *iutA* were found only among *K. pneumoniae* isolates. Significant differences in the prevalence of the genes were found between *K. pneumoniae*, *K. variicola,* and *K. quasipneumoniae* isolates as follows: *rmpA* (33.3% vs 0% vs 0%, *p* < 0.001), *iutA* (36.8% vs 0% vs 0%, *p* < 0.001), *ybtS* (47.1% vs 19.0% vs 0%, *p* < 0.001), *entB* (97.7% vs 90.5% vs 36.4%, *p* < 0.001), *kfu* (29.9% vs 90.5% vs 0%, *p* < 0.001), and *allS* (24.1% vs 4.8% vs 54.5%, *p* < 0.05). Significant differences in the prevalence of *rmpA, iutA*, *entB,* and *kfu* virulence factors were found even when the isolates were divided into hospital-acquired and community-acquired infection groups (Additional file 2: Table S3).

Of the 119 isolates, 16 (13.4%) belonged to the K1 serotype (*K. pneumoniae* = 15; *K. quasipneumoniae* = 1) and 13 (10.9%) to the K2 serotype (*K. pneumoniae* = 12; *K. variicola* = 1). Only 2 *K. pneumoniae* isolates (2.3%) and 1 *K. variicola* isolate (4.7%) were identified as ESBL producers (Additional file 2: Table S1). *K. pneumoniae* isolates belonging to K1 serotype had all 7 virulence factors, while *K. quasipneumoniae* belonging to K1 serotype had only the *allS* and *entB* virulence factors (Additional file 2: Table S1).

### Clinical characteristics

Of the 119 patients, 13 were excluded from the statistical analysis because of polymicrobial bacteremia. The characteristics of the patients are given in Table 2 and Additional file 2: Table S1. The most common underlying disease was malignancy (45 patients, 42.5%), which included both solid tumors and hematological malignant neoplasms. The most frequent source of bacteremia was abdominal infection (39 patients, 36.8%), which included liver abscess (7 patients 6.6%), urinary tract infections (31 patients, 29.2%), pneumonia (6 patients, 5.7%) and skin and soft tissue infections (4 patients, 3.8%). None of the patients had complications of endophthalmitis or meningitis in this study. Of the 119 patients, 56 (52.8%) had hospital-acquired infections. After the onset of bacteremia, 101 patients (95.3%) were treated with appropriate antibiotic therapy within 24 h. Eleven patients (10.3%) died during the first 30 days.

**Table 2:** Differences in clinical characteristics between patients with *K. pneumoniae*, *K variicola*, and *K. quasipneumoniae* bloodstream infections

Characteristics of patients	Total = 106		*K. pneumoniae* = 78		*K. variicola* = 19		*K. quasipneumoniae* = 9		*p*-value	
	n	%	n	%	n	%	n	%		
Age, median, years	74		72		81		77			
Age > 80 years	29	(27.4)	15	(19.2)	11	(57.9)	3	(33.3)	<0.005	*
Male sex	74	(69.8)	53	(67.9)	15	(78.9)	6	(66.7)	0.68	
Comorbidities			0.0							
Diabetes mellitus	29	(27.4)	20	(25.6)	7	(36.8)	2	(22.2)	0.61	
Malignancy	45	(42.5)	29	(37.2)	9	(47.4)	7	(77.8)	<0.05	*
Liver cirrhosis	11	(10.4)	9	(11.5)	2	(10.5)	0	(0)	0.87	
Collagen disease	8	(7.5)	8	(10.3)	0	(0)	0	(0)	0.36	
Chronic kidney disease	4	(3.8)	4	(5.1)	0	(0)	0	(0)	0.71	
Pulmonary disease	4	(3.8)	4	(5.1)	0	(0)	0	(0)	0.71	
Mental disorder	7	(6.6)	5	(6.4)	2	(10.5)	0	(0)	0.80	
Immunosuppressive drug	14	(13.2)	12	(15.4)	2	(10.5)	0	(0)	0.64	
Neutropenia	7	(6.6)	5	(6.4)	1	(5.3)	1	(11.1)	0.80	
Source of infection										
Pneumonia	6	(5.7)	5	(6.4)	1	(5.3)	0	(0)	1.00	
Skin and soft tissue	4	(3.8)	4	(5.1)	0	(0)	0	(0)	0.70	
Abdominal	39	(36.8)	27	(34.6)	9	(47.4)	3	(33.3)	0.40	
Urinary tract	31	(29.2)	26	(33.3)	2	(10.5)	3	(33.3)	0.14	
Liver abscess	7	(6.6)	5	(6.4)	2	(10.5)	0	(0)	0.79	
Others										
Hospital-acquired infection	56	(52.8)	41	(52.6)	10	(52.6)	5	(55.6)	1.00	
Appropriate antibiotic therapy within 24 h	101	(95.3)	75	(96.2)	17	(89.5)	9	(100)	0.52	

*Asterisk indicates a statistical significant difference among the groups

### Differences in clinical characteristics of bacterial species

The differences in the clinical characteristics among patients with *K. pneumoniae*, *K. variicola*, and *K. quasipneumoniae* infections are shown in Table 2. The median ages were 72, 81, and 77 years, respectively. *K. variicola* was the most frequent cause of BSI in patients over 80 years old (*p* <  0.005). BSIs with *K. quasipneumoniae* rather than *K. pneumoniae* and *K, variicola* (*p* <  0.05) frequently occurred in patients with malignancy. However, there were no significant differences in other characteristics (sex, source of infection, hospital or community acquired infection, or 30-day mortality) among patients with *K. pneumoniae*, *K. variicola,* or *K. quasipneumoniae* bacteremia. Regarding the phenotypes and prevalence of virulence genes, the hypermucoviscous phenotype was associated with intra-abdominal infection (odds ratio [OR] = 2.8; 95% confidence interval [CI], 1.0–8.2; *p* < 0.05) and liver abscess (OR = 26.7, 95% CI, 3.1–1335.6; *p* < 0.001). However, there were no significant differences in the prevalence of virulence genes and serotypes of isolates based on clinical characteristics (Additional file 2: Table S4).

### Differences in clinical characteristics between community-acquired and hospital-acquired infections

Of the 106 patients, 56 (52.8%) were categorized as having hospital-acquired infections. The differences in the clinical characteristics among patients with community and hospital-acquired infections are shown in Table 3. Malignancy was more frequently associated with BSIs in patients with hospital-acquired infections compared with community-acquired infections (53.6% vs 30.0%; *p* < 0.05). All ESBL-producing *K. pneumoniae* were found in only patients with hospital-acquired infections (2.8% vs 0%)*.* There were no significant differences in the other characteristics (sex, source of infection, prevalence of virulence genes, antimicrobial susceptibility, or 30-day mortality) between patients with hospital-acquired infections and those with community-acquired infections (Table 3 and Additional file 2: Table S4).

**Table 3:** Differences in clinical characteristics between patients with hospital-acquired and community-acquired bloodstream infections

Characteristics of patients	Total = 106		Hospital = 56		Community = 50		*p*-value	
	n	%	N	%	n	%		
Age, median, years	74		72		75.5			
Male sex	74	(69.8)	43	(76.8)	31	(62.0)	0.14	
Age > 80 years	29	(27.4)	12	(21.4)	17	(34.0)	0.19	
Comorbidities								
Diabetes mellitus	29	(27.4)	13	(23.2)	16	(32.0)	0.38	
Malignancy	45	(42.5)	30	(53.6)	15	(30.0)	< 0.05	*
Liver cirrhosis	11	(10.4)	7	(12.5)	4	(8.0)	0.53	
Collagen disease	8	(7.5)	3	(5.4)	5	(10.0)	0.47	
Chronic kidney disease	4	(3.8)	1	(1.8)	3	(6.0)	0.34	
Pulmonary disease	4	(3.8)	1	(1.8)	3	(6.0)	0.34	
Mental disorder	7	(6.6)	4	(7.1)	3	(6.0)	1.00	
Immunosuppressive drug	14	(13.2)	6	(10.7)	8	(16.0)	0.57	
Neutropenia	7	(6.6)	5	(8.9)	2	(4.0)	0.44	
Source of infection								
Pneumonia	6	(5.7)	1	(1.8)	5	(10.0)	0.10	
Skin and soft-tissue	4	(3.8)	2	(3.6)	2	(4.0)	1.00	
Abdominal	39	(36.8)	18	(32.1)	21	(42.0)	0.32	
Urinary tract	31	(29.2)	15	(26.8)	16	(32.0)	0.67	
Liver abscess	7	(6.6)	3	(5.4)	4	(8.0)	0.70	
Other								
Appropriate antibiotic therapy within 24 h	101	(95.3)	52	(92.9)	49	(98.0)	0.37	
Bacterial characteristics								
*K. pneumoniae*	78	(73.6)	41	(73.2)	37	(74.0)	1.00	
*K. variicola*	19	(17.9)	10	(17.9)	9	(18.0)	1.00	
*K. quasipneumoniae*	9	(8.5)	5	(8.9)	4	(8.0)	1.00	
K1 serotype	15	(14.2)	7	(12.5)	8	(16.0)	0.78	
K2 serotype	13	(12.3)	6	(10.7)	7	(14.0)	0.77	
Hypermucoviscous phenotype	23	(21.7)	9	(16.1)	14	(28.0)	0.16	
ESBL-producing	3	(2.8)	3	(3.4)	0	(0)	0.38	

*CRBSI* Catheter-related bloodstream infection

*Asterisk indicates a statistical significant difference among the groups

### Risk factor analysis for 30-day mortality

Of the 106 patients, 6 were excluded from the statistical analysis because we did not know the clinical courses of the patients during the 30 days following the onset of bacteremia. Risk factors found for 30-day mortality in univariate analysis were mental disorder (OR = 7.7; 95% CI; 1.0–59.0, *p* < 0.05) and skin and soft-tissue infection (OR = 9.2; 95% CI, 1.1–142.0; *p* < 0.05) (Table 4). However, there were no significant differences in bacterial characteristics, including species differences, between survivors and non-survivors. In multivariate analysis, liver cirrhosis (OR = 6.4; 95% CI, 1.0–37.0; *p* < 0.05), mental disorder (OR = 15.0; 95% CI,2.33–97.1; *p* < 0.005), and skin and soft-tissue infection (OR = 13.2; 95% CI, 1.19–145.0; *p* < 0.05) were found to be significant independent risk factors for 30-day mortality (Table 5).

**Table 4:** Univariate predictors of 30-day mortality among patients with bloodstream infections

Characteristics of patients	Total = 100		Fatal = 11		Survivors = 89		*p*-value	
	n	%	N	%	n	%		
Age, median, years	75.0		77.0		75.0			
Male sex	70	(70.0)	6	(54.5)	64	(71.9)	0.30	
Age > 80 years	27	(27.0)	4	(36.4)	23	(25.8)	0.48	
Comorbidities								
Diabetes mellitus	29	(29.0)	5	(45.5)	24	(27.0)	0.29	
Malignancy	41	(41.0)	3	(27.3)	38	(42.7)	0.52	
Liver cirrhosis	10	(10.0)	3	(27.3)	7	(7.9)	0.08	
Collagen disease	8	(8.0)	1	(9.1)	7	(7.9)	1.00	
Chronic kidney disease	3	(3.0)	1	(9.1)	2	(2.2)	0.30	
Pulmonary disease	4	(4.0)	1	(9.1)	3	(3.4)	0.38	
Mental disorder	7	(7.0)	3	(27.3)	4	(4.5)	< 0.05	*
Immunosuppressive drug	12	(12.0)	1	(9.1)	11	(12.4)	1.00	
Neutropenia	7	(7.0)	0	(0)	7	(7.9)	1.00	
Source of infection								
Pneumonia	6	(6.0)	0	(0)	6	(6.7)	1.00	
Skin and soft-tissue	4	(4.0)	2	(18.2)	2	(2.2)	< 0.05	*
Abdominal	37	(37.0)	3	(27.3)	34	(38.2)	0.74	
Urinary tract	27	(27.0)	2	(18.2)	25	(28.1)	0.72	
Other								
Hospital-acquired infection	52	(52.0)	6	(54.5)	46	(51.7)	1.00	
Appropriate antibiotic therapy within 24 h	96	(96.0)	11	(100.0)	85	(95.5)	1.00	
Bacterial characteristics								
*K. pneumoniae*	73	(73.0)	8	(72.7)	65	(73.0)	1.00	
*K. variicola*	19	(19.0)	2	(18.2)	17	(19.1)	1.00	
*K. quasipneumoniae*	8	(8.0)	1	(9.1)	7	(7.9)	1.00	
K1 serotype	14	(14.0)	3	(27.3)	11	(12.4)	0.18	
K2 serotype	13	(13.0)	0	(0)	13	(14.6)	0.35	
Hypermucoviscous phenotype	23	(23.0)	3	(27.3)	20	(22.5)	0.71	

*CRBSI* Catheter-related bloodstream infection

*Asterisk indicates a statistical significant difference among the groups

**Table 5:** Multivariate predictors of 30-day mortality among patients with bloodstream infections

	Odds ratio	95% CI	*p*-value	
Liver cirrhosis	6.43	1.0–37.0	< 0.05	*
Mental disorder	15.00	2.33–97.1	< 0.005	*
Skin and soft-tissue infection	13.16	1.19–145.0	< 0.05	*

*CI* Confidence interval

*Asterisk indicates a statistical significant difference among the groups

## Discussion

Previously, *K. variicola* and *K. quasipneumoniae* were often considered to be opportunistic pathogens with less virulence than *K. pneumoniae* in humans [14, 22]. However, recent studies have identified *Klebsiella* species on the basis of accurate molecular analysis and demonstrated that these pathogens are frequently detected at sites of infection in humans and in BSIs [15, 16]. In our study, *K. variicola* and *K. quasipneumoniae* were isolated from BSI patients without underlying disease, and surprisingly, some fatal cases of bacteremia were caused by *K. variicola* and *K. quasipneumoniae* (Additional file 2: Table S1). Taking these findings into consideration, it is possible that the pathogenicity of both *K. variicola* and *K. quasipneumoniae* has been underestimated.

Our study showed differences in the phenotypic features and the prevalence of virulence genes between *K. pneumoniae*, *K. variicola,* and *K. quasipneumoniae*. Among the phenotypic features, the hypermucoviscous phenotype was found not only in isolates of *K. pneumoniae* but also in those of *K. variicola* and *K. quasipneumoniae*. The presence of *rmpA* has been reported to be associated with the hypermucoviscous phenotype. However, in this study, *rmpA* was confirmed only in isolates of *K. pneumoniae* and could not be confirmed in those of *K. variicola* or *K. quasipneumoniae*. In fact, the presence of strains negative for the *rmpA* gene in hypermucoviscous phenotype isolates of *K. variicola* and *K. quasipneumoniae* has also been reported [23–25]. It was suggested that other mechanisms may contribute to the hypermucoviscous phenotype in these organisms.

In terms of virulence genes, significant differences in the prevalence of genes associated with iron-scavenging systems (*entB*, *ybtS*, *iutA*, and *kfu*) were recognized between *K. pneumoniae*, *K. variicola*, and *K. quasipneumoniae* isolates. The ability to acquire iron is essential for bacterial growth and replication, and the iron uptake system (*kfu*) contributes to invasive disease and could play a part in the pathogenicity of BSIs caused by *K. variicola* [10]. In our results, the presence of *kfu* was significantly higher in *K. variicola* isolates than in *K. pneumoniae* and *K. quasipneumoniae* isolates. It was previously reported that *K. pneumoniae* belonging to the K1 serotype strain has a higher prevalence of *kfu* than the non-K1 serotype [26, 27]. In this study, *K. pneumoniae* belonging to the K1 serotype carried *kfu* in all strains (16/16, 100%), whereas the K2 serotype had quite low rates (3/13, 23.0%). However, no *K. variicola* isolates were K1 serotypes. The carriage rates of *iutA* and *ybtS*, which produce aerobactin and yersiniabactin, respectively, were significantly higher in *K. pneumoniae* isolates than in *K. variicola* or *K. quasipneumoniae* isolates [9, 28, 29]. Moreover, *entB* was found in almost all *K. pneumoniae* and *K. variicola* isolates but only in 36.4% of *K. quasipneumoniae* isolates. These results suggested that *K. quasipneumoniae* isolates had fewer virulence genes associated with iron acquisition than *K. pneumoniae* or *K. variicola* isolates, as has been previously reported [8, 29–31]. On the other hand, the carriage of *allS*, which is associated with allantoin metabolism, was significantly higher in *K. quasipneumoniae* isolates (6/11, 54.5%) than in *K. pneumoniae* (21/87, 24.1%) or *K. variicola* (1/21, 4.8%) isolates. These results suggested that different mechanisms could contribute in part to the pathogenicity of BSI caused by three these organisms.

In this study, there were no significant differences between the three organisms in terms of clinical characteristics and impact on outcome, except for the fact that *K. variicola* was more frequently detected in elderly patients, and *K. quasipneumoniae* was found more frequently than *K. pneumoniae* and *K. variicola* in patients with malignancy in Japan. In comparing patients’ backgrounds between community-acquired and hospital-acquired infections, ESBL-producing isolates and patients with malignancies were more frequently associated with BSIs in the patients who had hospital-acquired infections*.* Indeed, these differences have been evaluated in previous reports; however, in most studies *K. variicola* and *K. quasipneumoniae* have unfortunately been misidentified as *K. pneumoniae* [32–35]. Our study showed that there was no significant difference in the rates of BSIs caused by the three types of *Klebsiella* species in patients with both community- and hospital-acquired infections.

Moreover, differences in bacterial properties and organisms were not identified as independent risk factors for 30-day mortality. One study showed that patients with *K. variicola* BSI have an increased risk of mortality compared with patients with *K. pneumoniae* or *K. quasipneumoniae* BSI in Sweden [19]. This contradiction probably reflects differences in bacterial populations due to the different geographical areas. In the Swedish study, there were fewer isolates belonging to K1 (1.4%) or K2 (5.0%) serotypes than in our study, which had 13.5% K1 serotype and 10.9% K2 serotype. K1 and K2 serotypes are frequently detected in BSIs, particularly in Asia and South Africa, but less frequently in Europe and North America [36]. Several reports have shown that K1 serotype strains, especially those belonging to clonal complex 23, have a high prevalence of plasmids encoding virulence genes, such as *rmpA*, *inuA*, *kfu* and *allS* [27, 37, 38]. Furthermore, such K1 *K. pneumoniae* strains are known as hypervirulent *K. pneumoniae*. There have been a few reports with detailed surveillance data on the distribution of capsular serotypes among *K. pneumoniae* isolates derived from patients with invasive infections across Japan [39]. Our study showed hypervirulent K1 *K. pneumoniae* is frequently the cause of BSI but is not associated with mortality; similar results have been found in Taiwan and China [40, 41]. It is reported that ESBL-producing isolates are associated with higher mortality rates [42]. However, in our study, ESBL-producing isolates were confirmed in only 3 of the 119 isolates. Therefore, depending on the geographical area, additional identification of *K. variicola* isolates from *K. pneumoniae* in routine laboratory tests may not help to lower the mortality rate of patients with BSI caused by *Klebsiella* species.

In this study, the limitations were the small sample size and low rate of mortality. A large sample size is needed to obtain better evaluation of accuracy, the prevalence of virulence genes, and further information on the differences in the clinical characteristics between *K. pneumoniae, K. variicola,* and *K. quasipneumoniae* infections.

## Conclusions

We identified *K. variicola* and *K. quasipneumoniae* using *parC* sequence-based analysis from isolates initially identified as *K. pneumoniae* obtained from patients with BSI. We found differences in the bacterial characteristics, including the prevalence of virulence genes and phenotypic features between these three phylogroups. BSIs caused by *K. variicola* were significantly more prevalent than those caused by the other two organisms in patients over 80 years old. However, there were no significant differences in 30-day mortality rates. Our findings provided evidence of the differences in bacterial pathogenicity and clinical features among these three phylogroups. Further research may help to determine additional clinical characteristics between these three organisms in patients with BSIs.

## Supplementary information

**Additional file 1: Figure S1**. Phylogenetic tree based on 314 bp *parC* gene of 119 *Klebsiella* clinical isolates and reference strains. The *parC* gene sequence of reference strains were imported from GenBank; *K. pneumoniae* strain ATCC 35657 (CP015134.1), *K. pneumoniae* strain ATCC 43816 (CP009208.1), *K. pneumoniae* strain SC-7 (CP030269.1), *K. pneumoniae* strain BAA-2146 (CP006659.2), *K. pneumoniae* strain NUHL 30457 (CP026586.1), *K. variicola* strain 13,450 (CP030173.1), *K. variicola* strain GJ3 (CP017289.1), *K. quasipneumoniae* strain ATCC 700603 (CP029597.1) and *E. cloacae* strain AR 0072 (CP026850.1). The phylogenetic tree was constructed by the neighbor-joining method and the reliability of the topology of each tree was checked by 500 bootstrap replications.

**Additional file 2: Table S1**. Summary of all clinical and bacterial characteristics in this study. **Table S2**. Differences in bacterial characteristics between isolates of hypermucoviscous and non-hypermucoviscous phenotypes. **Table S3**. Differences in in the prevalence of virulence genes and serotypes of isolates between hospital-acquired and community-acquired infection groups. **Table S4**. Differences in the prevalence of virulence genes and serotypes of isolates based on clinical characteristics.

## Data Availability

All data generated or analyzed during this study are included in this published article and supplementary files.

## Abbreviations

BSIs: Bloodstream infections
CLSI: Clinical and laboratory standards institute
ESBL: Extended spectrum beta-lactamase
MALDI-TOF MS: Matrix-assisted laser desorption ionization–time of flight mass spectrometry
*parC*: Subunit C of topoisomerase IV
