# Correction to: Clinical characteristics in blood stream infections caused by *Klebsiella pneumoniae*, *Klebsiella variicola*, and *Klebsiella quasipneumoniae*: a comparative study, Japan, 2014–2017

**DOI:** 10.1186/s12879-022-07384-5

**Published:** 2022-05-04

**Authors:** Kazuo Imai, Noriomi Ishibashi, Masahiro Kodana, Norihito Tarumoto, Jun Sakai, Toru Kawamura, Shinichi Takeuchi, Yoshitada Taji, Yasuhiro Ebihara, Kenji Ikebuchi, Takashi Murakami, Takuya Maeda, Kotaro Mitsutake, Shigefumi Maesaki

**Affiliations:** 1 Department of Infectious Disease and Infection Control, Saitama Medical University, 38 Morohongo, Moroyama-machi, Iruma-gun, Saitama, 350-0495 Japan; 2 Center for Clinical Infectious Diseases and Research, Saitama Medical University, 38 Morohongo, Moroyama-machi, Iruma-gun, Saitama, Japan; 3 Infectious Diseases and Infection Control, Saitama Medical UniversityInternational Medical Center, 1-1397 Yamane, Hidaka, Saitama 350-1298 Japan; 4 Clinical Laboratory Medicine, Saitama Medical University Hospital, 38 Morohongo, Moroyama-machi, Iruma-gun, Saitama, Japan; 5 Department of Clinical Laboratory Medicine, Saitama Medical University International Medical Center, 1-1397 Yamane, Hidaka, Saitama 1298 Japan; 6 Department of Microbiology, Saitama Medical University, 38 Morohongo, Moroyama-machi, Iruma-gun, Saitama, 350-0495 Japan

## Correction to: BMC Infectious Diseases (2019) 19:946 10.1186/s12879-019-4498-x

Following publication of the original article [1], the original PDF-publication of this article contained watermarking which indicated 'uncorrected proofs' whilst it was the final version.

The original article has been [1] updated to remove the watermarking.
