# Supplementary figures and images for: Clinical characteristics in blood stream infections caused by Klebsiella pneumoniae, Klebsiella variicola, and Klebsiella quasipneumoniae: a comparative study, Japan, 2014–2017
Source: BMC Infect Dis. 2019 Nov 8;19:946. doi: 10.1186/s12879-019-4498-x (PMC6842162; doi:10.1186/s12879-019-4498-x)

## Slide 1
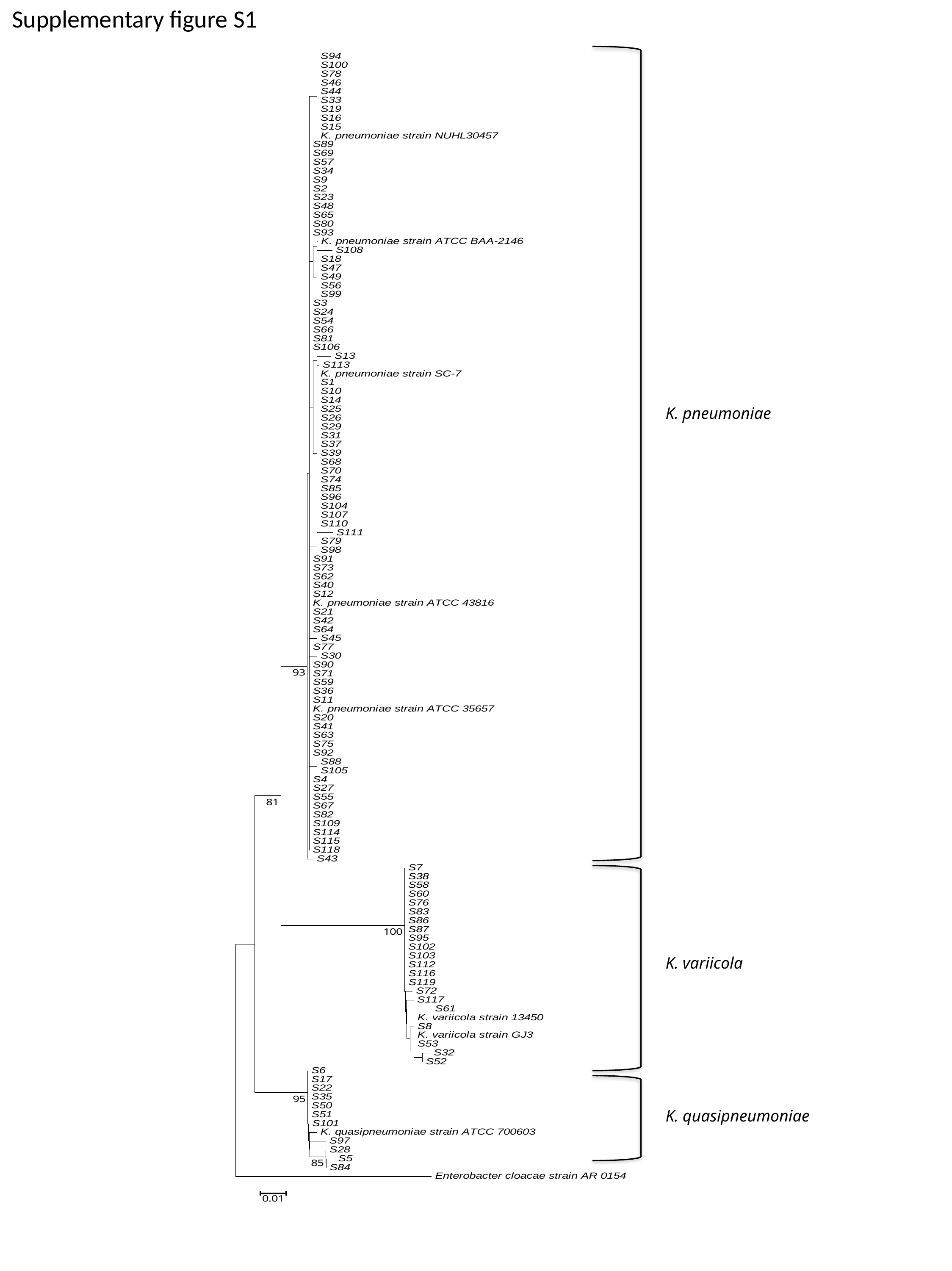

Supplementary figure S1
K. pneumoniae
K. variicola
K. quasipneumoniae

Supplement: Supplementary file 1 — Additional file 1: Figure S1. Phylogenetic tree based on 314 bp parC gene of 119 Klebsiella clinical isolates and reference strains. The parC gene sequence of reference strains were imported from GenBank; K. pneumoniae strain ATCC 35657 (CP015134.1), K. pneumoniae strain ATCC 43816 (CP009208.1), K. pneumoniae strain SC-7 (CP030269.1), K. pneumoniae strain BAA-2146 (CP006659.2), K. pneumoniae strain NUHL 30457 (CP026586.1), K. variicola strain 13,450 (CP030173.1), K. variicola strain GJ3 (CP017289.1), K. quasipneumoniae strain ATCC 700603 (CP029597.1) and E. cloacae strain AR 0072 (CP026850.1). The phylogenetic tree was constructed by the neighbor-joining method and the reliability of the topology of each tree was checked by 500 bootstrap replications. [file 12879_2019_4498_MOESM1_ESM.pptx]
